# Genome Sequence of Salmonella Phage χ

**DOI:** 10.1128/genomeA.01229-14

**Published:** 2015-02-26

**Authors:** Roger W. Hendrix, Ching-Chung Ko, Deborah Jacobs-Sera, Graham F. Hatfull, Marc Erhardt, Kelly T. Hughes, Sherwood R. Casjens

**Affiliations:** a Department of Biological Sciences, University of Pittsburgh, Pittsburgh, Pennsylvania, USA; b Biology Department, University of Utah, Salt Lake City, Utah, USA; c Pathology Department, Division of Microbiology and Immunology, University of Utah School of Medicine, Salt Lake City, Utah, USA

## Abstract

Salmonella bacteriophage χ is a member of the *Siphoviridae* family that gains entry into its host cells by adsorbing to their flagella. We report the complete 59,578-bp sequence of the genome of phage χ, which together with its relatives, exemplifies a largely unexplored type of tailed bacteriophage.

## GENOME ANNOUNCEMENT

The double-stranded DNA (dsDNA) lytic tailed bacteriophage χ was isolated in 1936 as a Salmonella enterica phage that infects flagellated cells only (1, 2). It has an isometric head of about 66 nm in diameter, a long noncontractile 230-nm tail (3), and a single 220-nm-long curly tail fiber that binds to the flagellum of its target cell (3–5).

Phage χ was grown on S. enterica, and its DNA was isolated and sequenced using previously described methods (6). The assembly of Sanger sequences of 384 randomly chosen clones generated six contigs with 17-fold redundancy. With 58 oligonucleotide primers on χ genomic template DNA, these were joined into a single contig, and all sequence ambiguities were resolved. The genome termini were determined by primer extension on genomic DNA to contain 12-base single-stranded DNA extensions with the sequence 5′-GGTGCGCAGAGC. The χ genome is 59,578 bp long (including one copy of the single-stranded sticky end at the left end) and has 56.5% G+C content; both are close to previously measured values (7). While this work was in progress, a supposedly complete χ genome sequence was reported by Lee et al. (8) (accession no. JX094499). However, that sequence is artificially joined at the cohesive ends and is missing 171 bp at the sequence ends (our bp 1 corresponds to bp 21230 in their sequence).

The phage χ genome contains 75 predicted protein-coding genes and no tRNA genes. The virion structure and assembly genes have a canonical organization, although the terminase subunit genes are displaced >10 kbp from the left *cos* end. The intervening region contains 10 genes, including DNA polymerase I, primase, nuclease, and two putative transcription regulator-encoding genes. Only three of the 34 predicted genes that lie to the right of the lysis genes have putative functions, encoding NinC-, DNA methylase-, and exonuclease-like proteins.

The recently released complete genome sequences of Salmonella phages iEPS5 (accession no. KC677662) (9), SPN19 (accession no. JN871591.1), and FSL_SP-030, FSL_SP-039, FSL_SP-088, and FSL_SP-124 (accession no. KC139519, KC139514.1, KC139512.1, and KC139515.1, respectively) (10), as well as those of Enterobacter cancerogenus phage Enc34 (11) (accession no. JQ340774) and Providencia stuartii phage RedJac (accession no. JX296113) (12) are quite similar in genome size, gene content, and gene order to those of the χ genome; their predicted major capsid proteins range from being 86% to 99% identical to that of χ. We note that most of these sequences are incomplete in that they are missing a few base pairs from the ends compared to the χ genome. These nine phages form a rather closely related group that is only very distantly related to other described phages, and we agree with Moreno Switt et al. (10) that they comprise a unique tailed phage type. We suggest this group be called the “χ-like phages” because χ was the first of the group to be isolated and studied. The Burkholderia phages AH2 (13) and BcepNazgul (accession no. AY357582) are the closest distant relatives of this group, and they carry syntenic DNA replication and virion assembly genes that are less similar to the χ genes (the major capsid proteins are 47% and 40% identical to χ).

### Nucleotide sequence accession number.

This sequence has been deposited in GenBank under accession no. KM458633.
